# Understanding Cross-Cultural Differences in Conceptualizing International Trade Patterns: A Neuroeconomic Perspective

**DOI:** 10.3389/fnins.2022.916084

**Published:** 2022-06-07

**Authors:** George Kwame Agbanyo, Yan Wang

**Affiliations:** ^1^School of Economics, Zhejiang University of Technology, Hangzhou, China; ^2^School of Business, Honghe University, Mengzi, China

**Keywords:** cognitive diversities, cross-cultural differences, neuroeconomics, neuroimaging, international trade patterns

## Abstract

Neuroeconomics has been seldom used in investigating the impact of culture on international trade. This research proposes a scientific approach to investigate how cross-cultural differences contribute to the conceptualization of international trade patterns globally. International business relations are directly influenced by factors such as cultural variations which distinguish one foreign market from another. Therefore, the level of understanding these cultural differences is able to determine the success or not of business opportunities. In response to the scarcity of scientific investigation of cultural influence on international trade, the purpose of this study is to propose a neuroeconomic framework as a strategic instrument to elucidate the cross-cultural dimension of international commercial relations. Echoing this, our study uses cultural diversities and cognitive classifications established in literature to adopt a unique scientific tool for the conceptualization of international trade patterns across the world. This research establishes the cognitive mechanism of cross-cultural diversity, as a novel framework to conceptualize international trade patterns. By unveiling the cognitive process of cross-cultural diversity, this article provides an instrument to unlock trade barriers of individualism and collectivism across nations.

## Introduction

The concept of economic integration among countries has long been demonstrated through classical economic theories and traditional frameworks of international organizations, whereby the role of cultural identity in the decision-making process had not been given a full attention (Shweder, 1991; Acheson and Maule, 2006; Podobas, 2011). But recent literature has explicitly proven how cultural differences constitute a remarkable barrier in business communication (Hofstede, 2000; Nowakowski, 2005; Chatterjee and Vartanian, 2016). Clearly, identifying and interpreting cultural differences is essential for establishing effective global business concepts in the areas of international trade communication, interactions, negotiations, management, marketing tactics, brand choices, and consumer behaviors (Nowakowski, 2005; Venkatesh and Van, 2005). Therefore, it is essential to clearly establish the implication of culture in the cognitive decision-making mechanism, and the nuances of cultural diversities on the neurological processes, especially for international business agreements. However, even though the influence of culture on the cognitive mechanism is well evidenced in literature, hitherto, the implication of such evidence on the business decision-making process in the brain has been explicitly documented (Nowakowski, 2005).

To fill this gap, this study proposes a neuroscientific analysis of cultural effects on the cognitive mechanism in international trade decision-making process. For a global trade contest, we employed neuroeconomic techniques to investigate further the effect of cross-culture diversity in the conceptualization of international trade patterns, thus to respond to the question:

1. How to conceptualize international trade patterns within a cross-cultural diversifies market?
2. What framework can provide a central scientific interpretation for such cross-cultural diversity in the decision-making mechanism?

We argue that for a complete understanding of the conceptualization of international trade patterns across nations/cultures, a neuroscientific examination of the cultural effect on the cognitive mechanism is imperative (Camerer et al., 1989, 2005). This article contributes to literature, first by adopting a neuroscientific approach to elucidate the intricate cognitive mechanism resulting from cultural implications in the business decision-making process. Secondly, taking international trade setting as a perfect cross-cultural environment, this study scientifically demonstrates the implication of cross-cultural diversities on the conceptualization of international trade patterns. In this regard, this article conceives an interdisciplinary framework to merge culture, economics, and neuroscience, in establishing consensual patterns adaptable for business establishments across nations.

## A Review of International Trade Concepts Across Cultures

Defined as the exchange of capital, goods and services across territories, international trade is a complex process due to the numerous differences between patterns such as currency, government policies, judicial systems, laws, and markets influence trade (Hofstede, 1980; Cheng et al., 2022; Chin et al., 2022). But to easy trade between countries, international economic organizations were formed to establish concepts that promote uniform market conditions for members, mostly based on traditional economic determinants (GDP, tax, comparative advantage, and resource endowment) (Yukl, 1994; Venkatesh and Van, 2005). Unfortunately, social and cultural implications to shaping the patterns of international associations, as relevant as are to the success or not of international relations, have not been given the necessary attention especially in research discussions (Cheng et al., 2022). Meanwhile, geographical distribution of culture is an unavoidable issue in international trade where cultural distance is considered as a fundamental element of analyzing trade decisions (Hofstede, 1980; Acheson and Maule, 2006). Although, some researchers like Chin et al. (2021) argue that distance is irrelevant in the advanced ICT era, the perspective in this discussion supports the view that cultural distance still has an important interference in international trade since technology can not completely nullify its effect. As defined by Vartanian et al. (2015), cultural distance refers to the set of factors creating obstacles to the knowledge flow, and in trade, between the source and target destination.

As in this context, cultural diversity creates a barrier to the transfer of knowledge, information, and competencies and ultimately the cost of doing business (Perano et al., 2018; Chin et al., 2022). According to Hofstede (1980), distance directly determines the kinds of partners one can attract. He posits that investors prefer to invest in culturally close countries instead of culturally distant ones. Taking together the foregoing arguments, the gravity model one of the most adopted economic models that explains bilateral transfers based on economic size and distance (geographical, cultural, etc…) between two partners. There is a rising tendency for technology advantages to wear off, causing rivalry to move toward economic and cultural closeness (Hofstede, 1980). The author explained that is a compelling case for including cultural concerns into strategic planning and situating operations in nations and organizations that have the cultural qualities required to compete in these activities.

As well documented in literature, international trade decision-makings process has been totally attributed to market parameters based empirical research. However, the implication of cultural parameter pushes the discussion to take a broader dimension, where the decision-making process could be subjected to a cognitive scientific experimentation (Camerer et al., 1989; Quan et al., 2021). Consequently, the novel interdisciplinary field of neuroeconomics emerged, with the focus of investigating the neural mechanism in an economic decision-making process (Camerer et al., 1989, 2005; Glimcher, 2002, 2003). Echoing this, the novel interdisciplinary field, bases on neuroscientific findings to investment the process of cognitive reaction to cultural parameters. So far, research has traced neural stimuli response to cultural activities, nevertheless the inference of culture on the cognitive mechanism and their effect on the economic decision-making process is not elaborate in literature (Glimcher, 2003; Camerer et al., 2005). As already established, on one hand, culture plays an important role in the economic decision-making process, on the other, the neuroscientific techniques reveal the cognitive stimuli reaction to cultural activities, but there is lack of neural evidence of cultural implication of the economic decision-making mechanism. Therefore, the neuroscientific evidence of cultural influence on the economic decision-making is instrumental for the conceptualization of international trade patterns.

## International Trade Patterns Between People From Diverse Cultures

As established above, people’s perspective and reaction in international trade is directly influenced by their cultural background. In terms of the attitudes toward relations and business, literature has distinguished categorizations setting where cognitive mechanism is contrasted and compared to cultural identity (Hofstede, 1980; Sanfey et al., 2003; Acheson and Maule, 2006; Podobas, 2011). For instance, a pairing between transaction-oriented and relationship-oriented cultures is considered as a relevant determinant in business negotiations. The main objective of transaction-oriented cultures is to conclude the transaction, gain profit with little or no attention to the relation with the trading partner. But the relationship-oriented cultures rather pay attention to establishing and maintaining good relations between people, with the transaction itself taking the second place (Sanfey et al., 2003; Hofstede et al., 2010). Another setting established on the point of view of the manner in which people behave, has distinguishes reserved and expressive cultures. Expressive culture people easily show their emotions and feelings, using a range of non-verbal means. But in reserved cultures, emotions and feelings are hardly expressed. In terms of the attitude toward time, the monochronic and polychronic cultures are the most recognized in literature (Hofstede, 2000; Podobas, 2011).

Further classification of cultural backgrounds in typical negotiation situations could also be based on country differences. The Asian culture on the other side is characterized by a cultural and religious affiliation, with an adherence to tradition. The key to success is collectivism where, every stage of the negotiation process, the consent of the entire group is necessary. The Asian culture is a reserved culture, relationship-oriented, polychronic, and formal. When conducting negotiations, either successful or not, “saving face” is extremely important for the Asians. Relations between parties should be reserved in showing their feelings and emotions (Podobas, 2011). The United States is one of the largest economies in the world, with this country’s companies making businesses virtually with every other country and marking their presence on most foreign markets is characterized by a first contact greeting accompanied by eye contact. Regarding business meetings, Americans like straight negotiations, without prolonging talks. This concept is basically founded on the following their famous motto “time is money” and concluding a negotiation by signing an agreement is paramount (Duan et al., 2021; Huang et al., 2022). The European countries are much more diverse culturally, mainly divided into three major groups; the Mediterranean countries, the north of Europe, and the central part of Europe. There is no single characteristic feature for all the European countries (Sanfey et al., 2003; Podobas, 2011). This dissertation presents the following countries for this discussion: France, Germany, and the United Kingdom with each of them located in a different part of Europe, therefore each having a different culture. The Mediterranean countries represented by France are characterized by an expressive culture, moderately transaction-oriented, formal and moderately monochronic. In France, contacts are crucial. They much care about their own interests first or take advantage of any oversight. Germany represents another important cultural grouping in Europe with the strongest economy. The handshake should be firm and cordial. Punctuality is crucial to Germans, and they highly value organization of work and order with precision and responsibility. They are almost always perfectly prepared. The United Kingdom represents another cultural setting clearly characterized by a monochromic cognition, moderately formal, reserved and transaction-oriented. In United Kingdom, punctuality, competence and discipline are valued (Sanfey et al., 2003). They are sincere and diplomatic. They do not tolerate a lack of competence, unreliability, and aggressive behavior. The Middle East is comprised in majority of Arabs countries, with an expressive, formal, and relationship-oriented and polychronic culture. Critical remarks, even those which seem entirely insignificant are to be avoided (Costa-Gomes and Crawford, 2006). While Arabs people themselves do not observe timeliness and punctuality is valued on the part of counterparties (Duan et al., 2021; Huang et al., 2022; Sanfey et al., 2003). These cultural characteristics and the geographical identities are summarized in Table 1.

**TABLE 1:** Cognitive classifications of cultural diversities.

Types of culture	Cognitive ethical characteristics	Countries within a cultural setting
Transaction-oriented cultures	Concentrate on goal achievement, little attention on relation with partners but on transaction and business profit.	Scandinavian, North America, Australia, New Zealand, United Kingdom, South Africa, Central and Eastern Europe, Brazil, Hong Kong.
Relationship-oriented cultures	Relationship oriented cognitive, emphasis on human relationship, transaction takes second place.	Arab countries, Most African, Asian and Latin American countries.
Informal culture	Less attention to social hierarchy, superiors are considered as older colleagues, small difference in social status.	Australia, New Zealand, North America, Scandinavian countries.
Formal culture	Social status is very relevant, great respect toward the elderly and more experienced.	Majority of Asian, European and Latin American countries.
Reversed	Quiet and calm in negotiations, do not pay much attention to close or eye contact, do not reveal emotions.	South and South-East Asia, Latin America, Germanic countries of Europe.
Expressive	Emotional expression, non-verbal expression, touch and eye contact.	Romance European countries, Mediterranean countries, Latin America countries.
Varying degrees of expression	In between the reversed and expressive cultures.	Eastern Europe, South-Asia, African countries, Poland.
Monochronic	View time as linear and an essential non-renewable resource, punctuality and accuracy are paramount, clearly separate work and private life, highly value breaks and personal times.	Normandy and Germanic European countries, North American countries.
Polychronic	Meeting tine is flexible; less emphasis to punctuality, the main topic of a conversation can be departed from, no need to rush people.	The Arab world, most of African and Latin America countries, South and South-East Asia.

*Source: Hofstede (1980), Sanfey et al. (2003), and Podobas (2011).*

**FIGURE 1:**
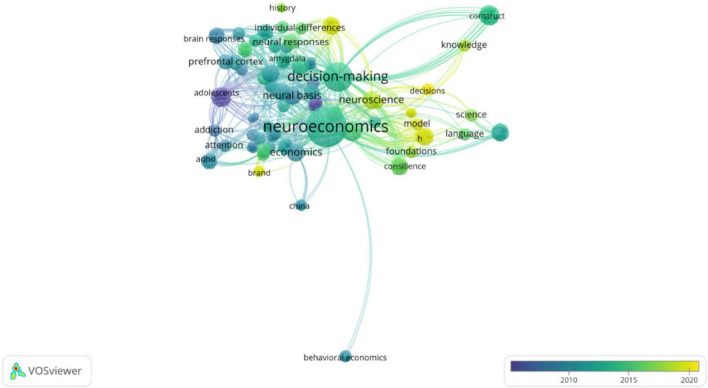
Scientometrics analysis of “Neuroeconomics and culture” for 2003–2022. Source: VOSviewer interpretation from web of science data.

To conclude, so far, there is no consensus on the patterns of international trading as they reflect different meanings and ways of doing business across cultures (Hofstede, 2000; Hofstede et al., 2010). From this systematic analysis, business transaction (Pellicano et al., 2016; Casali and Perano, 2020) is interpreted from diverse cognitive orientations strongly anchored on the cultural foundations on which people function. Africans, Americans, Asians, or Europeans clearly present different cognitive identities; moreover, each continent also represents behavioral reactions underpinned by a cultural diversity. Furthermore, the perspective of this dissertation is to deepen the cognitive underpinning of cross-cultural differences on conceptualizing the notion of international trade from a novel angle of neuroeconomic perspective. From a scientometric analysis of “neuroeconomic*” “Cultur*” from Web of Science database, we observed that since 2003 up to date, the interdisciplinary field of neuroeconomics has merged different subjects but the cultural effect on the decision-making mechanism has not been well documented (Figure 1).

## International Trade Patterns From a Neuroeconomic Perspective

Understanding the cognitive diversities across cultures, especially in the uncertain market, provides an assurance for successful results, as compared to a mere reliance on the classical economic parameters of market demand and supply. Indeed, an indebt examination of the neural interpretation of cultural diversities, and for this discussion very instrumental in bridging fundamental barriers of international trade (Shweder, 1991; Nowakowski, 2005). Obviously, with the fast rate of globalization, comes more compounding challenges, therefore a neuroeconomic interpretation of cultural diversity needs more attention for concrete conceptualization of international trade patterns (Sanfey et al., 2003). Nevertheless, neuroeconomics being an emerging subject calls for more research, especially the cross-cultural diversity implication in international trade patterns. In the next lines, we explore the highlights of classical economic evolution until the introduction of neuroscience parameters into the economic discussion in Glimcher (2003), Camerer et al. (2005).

Founded on market parameters like demand and supply, the birth of classical economics was basically driven by rational and unemotional players with a focus on profit maximization. However, after a century-long detour, the recognition of psychological behaviors in the economic decisions making process opened up a new neoclassical era of economics traceable to Smith’s (1776) master pieces “The invisible hand” and “The wealth of nations.” At this stage, economics demonstrated the basic psychological underpinning of economic outcomes. Further research finding the link between individual behaviors to their cultural background demonstrates the intrinsic correlation between culture and the cognitive process of an individual’s brain, also referred to as the psychology of homo-economicus. Along this line of thought, further research attempt to conduct a deeper probe into the neural mechanisms in relation to cultural diversity and its impact on economic decisions, giving birth to the neuroeconomic field of study (Glimcher, 2003; Camerer et al., 2005). Neuroscience is responsible to explain further, by brain mapping, the neural reactions at different regions of the brain in relation to cultural identity and economic decisions (Glimcher, 2003). In the perspective of this research, we consider the benefits of further studies on the dynamics of culture and its role in the decision-making patterns in terms of international trade, since such transactions operate in diversified cross-cultural communities.

In this discussion, we emphasize the impact of cross-cultural diversity on the conceptualization of international trade patterns. Obviously, cognitive difference across cultures does not facilitate a consensual trade pattern adapted for economic transactions across nations. Therefore, we propose a deeper analysis of the situation from a neuroscientific perspective, in order to analyze the functions of the brain in correlation with cultural identities and adapted business patterns. Moreover, we posit that, even with the trade barriers of individualism and collectivism across nations, from a neuroeconomics perspective, international trade patterns could be developed in correspondence with specific cultural identities (Chatterjee and Vartanian, 2016). Based on Camerer et al. (2005) findings, new functional magnetic resonance imaging (fMRI) and transcranial magnetic stimulation (TMS) techniques in neuroscience have become instrumental in identifying the different regions of the human brain and the corresponding neural reaction in the decision-making process. Neuroimaging techniques including electro-encephalography (EEG) are even used to capture images of the brain to determine diverse neural reactions from coloration and electrical activity differentiations of different regions of the brain (Glimcher, 2003). A similar and complementary technique, the magnetoencephalography (MEG) and the positron emission tomography (PET), can determine much more sensitive magnetic field and blood flow in specific portions of the brain which can interpret the neural activities in response to specific decision-making process traceable to cultural identification (Glimcher, 2003; Camerer et al., 2005). Especially, this article endeavors to propose the adoption of a neuroeconomic approach in the conceptualization of international trade patterns across nations. Obviously, with the neuroscientific discoveries, the correlation between cultural identities and corresponding neural reactions could facilitate the conceptualization of international trade patterns favorable to specific cultural identities (Vartanian et al., 2015; Cheng et al., 2022).

## Conclusion and Further Research

Even though classical economic theories were instrumental in laying the foundations for the economic understanding, they became inadequate to explain broader determinants in the interpretation of the economic decision-making process. For instance, the implication of cultural diversity in the international trade procedures, need further expansion of the economic framework to include cognitive parameters (Hofstede, 1980). Consequently, the exploration of international trade patterns across nations is fundamentally resourced from a multidisciplinary integration, giving rise to nascent research fields of neuroeconomics. Introduced just a little more than a decade ago, neuroeconomics provides the perfect framework for examining the function of the brain in response to economics decision making, especially with the interference of cross-cultural diversities (Camerer et al., 2005). However, the implication of cultural diversity on the cognitive mechanism, as relevant as it is to the international trade conceptualization, has not been adequately explored. To establish a firm foundation for the conceptualization of international trade patterns, this dissertation, through a neuroeconomic lens, explore the intricate stimulations of the brain in relation to cultural identification on the economic interactions across nation (Glimcher, 2003). With recent discoveries in neuroscience like neuroimaging techniques, the neural metabolisms are largely identified by the reaction of different parts of the brain, as a result of cultural identification in close relations with the economic decision-making process (Glimcher, 2003; Camerer et al., 2005). Indeed, new discoveries in neuroscience are very instrumental for the conceptualization of international trade patterns, whereby trade transactions among foreign partners is largely determined by the cognitive diversities across cultures. Therefore, this study demonstrates the complexity in the conceptualizing of a consensual international trade pattern, therefore this discussion proposes a neuroeconomic perspective to consolidate an adapted international trade pattern to specific cultural identities. Being a novel research field, neuroeconomics needs further examinations. Future research can investigate the international trade patterns identical to economic sectors and further explore corresponding cognitive patterns identical to each sector across cultures. Determining such sectorial cognitive identities with a corresponding international trade pattern across nations promises a great step toward a successful international trade according to each particular economic sector even in an unstable market.

## Data Availability Statement

The original contributions presented in this study are included in the article/supplementary material, further inquiries can be directed to the corresponding author.

## Author Contributions

GA designed and conducted the research and wrote the first draft of the manuscript. YW searched and analyzed the literature and wrote the main part of the manuscript. Both authors contributed to the article and approved the submitted version.

## Conflict of Interest

The authors declare that the research was conducted in the absence of any commercial or financial relationships that could be construed as a potential conflict of interest.

## Publisher’s Note

All claims expressed in this article are solely those of the authors and do not necessarily represent those of their affiliated organizations, or those of the publisher, the editors and the reviewers. Any product that may be evaluated in this article, or claim that may be made by its manufacturer, is not guaranteed or endorsed by the publisher.
